# Rapid detection of *Staphylococcus aureus* using a novel multienzyme isothermal rapid amplification technique

**DOI:** 10.3389/fmicb.2022.1027785

**Published:** 2022-10-13

**Authors:** Pengfei Heng, Jiakai Liu, Zhen Song, Chuan Wu, Xiuzhong Yu, Yang He

**Affiliations:** ^1^State Key Laboratory of Southwestern Chinese Medicine Resources, College of Medical Technology, Chengdu University of Traditional Chinese Medicine, Chengdu, Sichuan, China; ^2^Department of Ultrasound Medicine, The Second Affiliated Hospital of Chengdu Medical College, China National Nuclear Corporation 416 Hospital, Chengdu, Sichuan, China; ^3^Department of Laboratory Medicine, People’s Hospital of Xinjin District, Chengdu, Sichuan, China

**Keywords:** multienzyme isothermal rapid amplification, *Staphylococcus aureus*, rapid detecting, fluorescence, diagnosis

## Abstract

*Staphylococcus aureus* is a common pathogen that causes various infections. Therefore, it is crucial to develop a fast and easy detection method for diagnosing and preventing *S. aureus* infections. In this study, MIRA assay was developed and validated (specificity; 100%) for the detection of *S. aureus* with *nuc* as the target gene. The reaction temperature and reaction time were then optimized, and the best reaction was at 40°C, 20 min. The assay could detect *S. aureus* in only 25 min. Additionally, the limit of detection of MIRA was 5 × 10^2^ CFU/ml, 10-fold lower than that of the traditional PCR. Furthermore, this assay efficiently detected 219 *S. aureus* of 335 strains obtained from different bacterial samples (detection accuracy; 99.40%). In conclusion, this study provides a rapid and easy-to-operate method for the detection of *S. aureus*, and thus can be used for the timely diagnosis and prevention of *S. aureus* infection.

## Introduction

*Staphylococcus aureus* is one of the most common pathogens in clinics causing various infections (Van Wamel, 2017; Oliveira et al., 2018), such as enteritis, pneumonia, endocarditis, and bacteraemia (Parker and Prince, 2013; Shorr et al., 2015; Kimmig et al., 2021). With strong virulence, invasiveness, and antibiotic resistance, *S. aureus* has become the primary pathogen of hospital and community-acquired infections (Long et al., 2022). Furthermore, *S. aureus* resistance has gradually increased because of the long detection time and antibiotic misuse (Mu et al., 2016; Shrestha et al., 2019). In particular, *methicillin-resistant Staphylococcus aureus* has become one of the most crucial epidemiological problems in hospitals worldwide, resulting in a large number of premature deaths (Pottinger, 2013). Therefore, a rapid method should be developed for the accurate screening of *S. aureus* infections to enhance prevention and treatment.

The current standard clinical methods for the isolation and identification of *S. aureus* depend on culture and microscopy (Zhao et al., 2016). Although these methods are stable, inexpensive, and can give both qualitative and quantitative information on the number and the nature of micro-organisms present (Long et al., 2022), they are time-consuming and the complicated operation, thus not suitable for timely diagnosis and treatment. The process, from sample collection, selective culture, and biochemical screening and serological confirmation, takes about 2 to 5 days. However, polymerase chain reaction (PCR) has significantly improved bacterial detection because of the advancement in molecular diagnostic technology (Kim et al., 2001; Huletsky et al., 2004). PCR detection method takes about 2–3 h. Recent studies have developed new and efficient detection methods for *S. aureus*, such as loop-mediated isothermal amplification (LAMP; Sowmya et al., 2012), recombinase polymerase amplification (RPA)/recombinase-aid amplification (RAA; Du et al., 2018; Geng et al., 2018), chromatographic methods (Zhou et al., 2022), immunological methods (Zhao et al., 2020) and aptamer-based methods (Yuan et al., 2014; Shahdordizadeh et al., 2017). Fortunately, these technologies are capable of detecting low counts of bacterial cells (Cabicarová et al., 2016). However, the widespread application of these techniques is limited by the complex instrumentation, complicated procedures, high instrument costs, and time-consuming (Sowmya et al., 2012; Wu et al., 2016). Therefore, it is necessary to develop a rapid, simple, sensitive, and specific detection method for *S. aureus*.

Isothermal amplification technology is a nucleic acid *in vitro* amplification technology, and the reaction process is always carried out at a constant temperature. The purpose of rapid amplification is achieved by adding enzymes with different activities and various specific primers to the reaction system (Yan et al., 2014). One of them is Multienzyme Isothermal Rapid Amplification (MIRA), which achieves rapid nucleic acid amplification at room temperature based on the synergistic action of various functional proteins (helicase, recombinase, single-stranded binding protein, DNA polymerase). Unlike RPA (T4 UvsX) and RAA (*E. coli* UvsX), MIRA uses a different source of a recombinant enzyme (*Streptomyces azure* recA, SC-recA) to improve the stability of the reaction and interference resistance. The reaction is usually completed within 30 min at 37–42°C, depending on the experiment. Agarose gel electrophoresis, real-time fluorescence, and lateral flow strip assay can be used to analyze MIRA results. Although several studies have used the MIRA technique to detect various infectious diseases, such as SARS-CoV-2 (Chen et al., 2021), *Mycobacterium tuberculosis* (Lu et al., 2021), and hepatitis B (Sun et al., 2021), this method has not been used to detect *S. aureus*. Therefore, we hope to establish a rapid detection of *S. aureus* through this method.

In this study, a MIRA-based assay was established for the rapid, specific, and sensitive identification of *S. aureus* by targeting the *nuc* gene (Brakstad et al., 1992). The feasibility of the MIRA experiment was verified by the detection of the collected clinical samples. Furthermore, on the application of MIRA to the clinical samples, we found that MIRA has higher specificity than PCR. This method may provide a more rapid, accurate, and suitable method for detecting *S. aureus* in clinics.

## Materials and methods

### Bacterial strain and genomic DNA extraction

A total of 12 strains (1 *S. aureus*, 2 *Staphylococcus* strains, and 9 non-*Staphylococcus* spp. strains) were used in this study to analyze the specificity of the MIRA reaction as shown in Table 1. Strains (335) were obtained from patients admitted to the Traditional Chinese Medicine Hospital of Sichuan, the Fifth People’s Hospital of Chengdu, and People’s Hospital of Xinjin District. The 12 strains were cultured in Trypticase Soy Broth (TSB) at 37°C and 200 rpm overnight. Bacterial DNA Extraction Kit (Magen Biotech Co., Ltd) was used for genomic DNA extraction. DNA concentration was determined using an ND5000 ultra-micro UV–Vis spectrophotometer (Bioteke Corporation), then stored at −20°C.

**Table 1:** Bacterial strains used in this study and results of analytical specificity of MIRA assays.

Bacteria	Source of strain	No. of strains	MIRA results
*Staphylococcus* spp.			
*S. aureus*	ATCC 25923	1	P
*S. haemolyticus*	ATCC 29970	1	N
*S. epidermidis*	ATCC 12228	1	N
Non-*Staphylococcus* spp.			
*Enterobacter cloacae*	CMCC(B) 45301	1	N
*Enterococcus faecalis*	ATCC 29212	1	N
*Enterococcus Faecium*	ATCC 19434	1	N
*Escherichia coli*	ATCC 25922	1	N
*Klebsiella Pneumoniae*	CMCC(B) 46117	1	N
*Vibrio parahemolyticus*	ATCC 17802	1	N
*Shigella dysenteriae*	CMCC(B) 51105	1	N
*Salmonella enterica*	ATCC 14028	1	N
*Pseudomonas aeruginosa*	ATCC 27853	1	N

ATCC, American Type Culture Collection; CMCC, National Center for Medical Culture Collections; P, positive result; N, negative result.

### Primer and probe design

Primer length of 30–35 bp and amplicon lengths not exceeding 500 bp are recommended for the MIRA reaction. Herein, specificity analysis of the three designed primer primers was conducted using the BLAST tool. The *nuc* gene (GenBank Accession No. EF529608) was downloaded from the NCBI GenBank database. The probe sequence had four modification sites: a dSpacer (THF) at the middle of the 5′ end as a nucleic acid exonuclease recognition site, a fluorescent group (FAM) upstream, a quenched group (BHQ) downstream, and a C3-Spacer at the 3′ end. All primers and probes were synthesized at Sangon Biotech Co., Ltd. (Shanghai, China).

### Optimization of temperature and time in MIRA reaction

MIRA experimental reactions were conducted using amplification kits from AMP-Future Biotech Co. Ltd. The mixture was prepared in a tube containing buffer A (29.4 μl), 2 μl (10 μM) of upstream primer, 2 μl (10 μM) of downstream primer, 0.6 μl (10 μM) of probe, 11.5 μl of ddH_2_O and 2 μl of extracted genomic DNA template. The 2.5 μl B buffer was added up to the tube cover, then the tube was shaken up and down to mix, and next MIRA reactions were performed on real-time fluorescence quantitative PCR (Analytik Jena AG, Jena, Germany) under optimized conditions. Positive results in the experiment will show a clear amplification curve, negative results will not.

The effect of different temperatures (37°C, 38°C, 39°C, 40°C, 41°C, and 42°C) and reaction times (5, 10, 15, 20, 25, and 30 min) on the MIRA reaction was explored to determine the optimum conditions. First add the amplification kit and template to the same tube, invert up and down for 7–8 times and mix thoroughly and mixed thoroughly, and finally the reaction solution was quickly centrifuged at 1000 rpm for 15 s. A qRT-PCR was used to conduct the MIRA, and FAM channel fluorescence values were collected every 15 s. The real-time fluorescence data obtained at different temperatures were analyzed to obtain the final optimum reaction temperature. Finally, the shortest reaction time under the optimized temperature was determined as described above.

### Specificity analysis

The specificity of the MIRA reactions was evaluated using 12 strains, including three *Staphylococcus* strains and nine non-*Staphylococcus* strains. Genomic DNA was extracted from the strains using Bacterial DNA Extraction Kit. MIRA amplification reaction was conducted under the optimized reaction system. *S. aureus* (ATCC 25923) and distilled water were used as positive and negative control, respectively. The amplified results were observed using qRT-PCR. The MIRA reaction solution was digested using Proteinase K at 37°C for 15 min, centrifuged for 5 min, and the product was analyzed by 1.5% agarose gel electrophoresis to further validate the specificity of the amplification products. The experiments were repeated thrice.

### Sensitivity analysis

The standard *S. aureus* (5 × 10^8^ CFU/ml) cultured in TSB was serially diluted 10 times to obtain nine bacterial suspensions (5 × 10^8^–5 × 10^0^ CFU/ml) to determine the limit of detection for the MIRA reaction. Genomic DNA was extracted at various concentrations. MIRA reactions were then conducted under optimized conditions. The limits of detection between the MIRA and PCR methods were also compared. Nine concentrations of genomic DNA were simultaneously subjected to PCR reactions using the same primers used in the MIRA and 2 × EasyTaq® PCR SuperMix (TransGen Biotech Co., LTD). The PCR reaction conditions included: pre-denaturation at 94°C for 5 min, denaturation at 94°C for 30 s, annealing at 55°C for 30 s, extension at 72°C for 45 s, 35 cycles, and extension at 72°C for 5 min. The experiments were repeated thrice.

### Detection of clinical samples and data analysis

A total of 335 clinical specimens (Table 2) were inoculated on blood agar plates, and single colonies were isolated from the plates, sample types included sputum, blood, secretions, ascites, abscess puncture fluid, and urine. The strains were cultured in TSB at 37°C and 200 rpm overnight. Use chemical lysis method to obtain genomic DNA, next mixing with MIRA reagent, the reaction was carried out at 40°C for 20 min, and the positive result was identified by amplification curve. The strains were identified using the clinical VITEK-2 Compact (Biomerieux, France) fully automated microbial identification system for the diagnostic evaluation of MIRA and PCR experiments. In addition, all clinical samples were assayed using PCR. All experiments were repeated thrice. The efficacy of MIRA and PCR was analyzed based on sensitivity, specificity and accuracy as follows: Sensitivity = TP/(TP + FN) × 100%; Specificity = TN/(TN + FP) × 100%; and accuracy = (TP + TN)/(TP + FP + TN + FN) × 100% (TP, true positive; FP, false positive; TN, true negative; FN, false negative).

**Table 2:** Information on clinical isolates (*S. aureus* strains and non-*S. aureus* strains).

No. of strains	Sample type	Clinical diagnosis		MIRA results	
		Positive	Negative	Positive	Negative
71	Sputum	34	37	33	37
59	Blood	46	13	46	13
109	Secretions	88	21	88	21
17	Ascites	13	4	13	4
29	Abscess puncture fluid	23	6	23	6
50	Urine	17	33	16	33

## Results

### Primer screening

**Figure 1:**
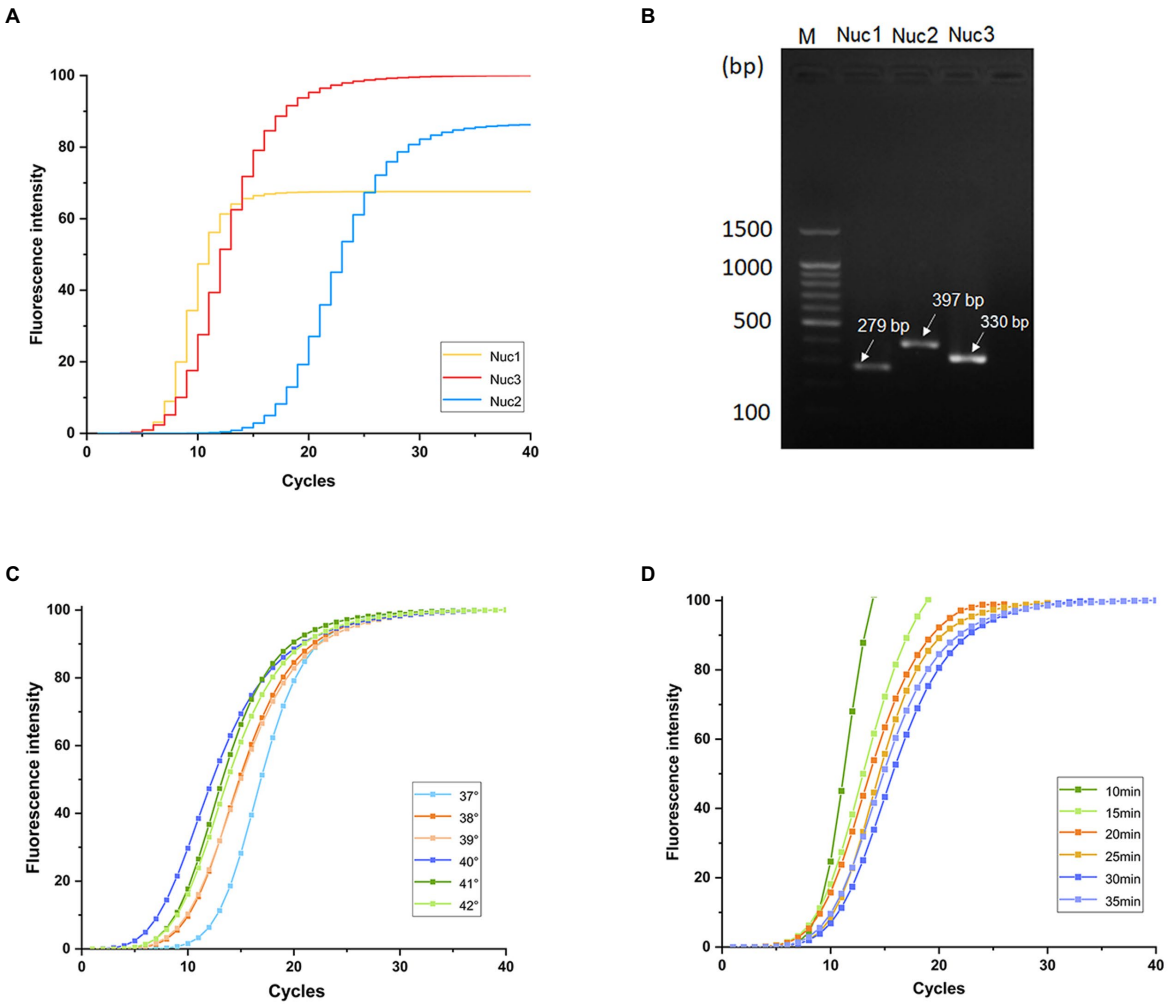
Primer screening and optimization of MIRA temperature and time. **(A)** Fluorescence intensity values of three pairs of primers. **(B)** Band diagram of three pairs of primers agarose gel electrophoresis analysis results. Optimization of MIRA reactions at different temperatures **(C)** and times **(D)**.

Fluorescence intensity values of qRT-PCR, as an evaluation indicator, showed that *nuc*3 was the most efficient primer based on the amplification curves (Figure 1A). Furthermore, agarose gel electrophoresis showed that the electrophoretic bands of the three target primer pairs were consistent with the size of the expected amplification products. The *nuc*3 primer (330 bp) had the brightest electrophoretic band (Figure 1B). The *nuc*3 gene sequences are shown in Table 3. These findings indicate that the *nuc*3 primer is efficiently amplified in the MIRA reaction compared with the other two primer pairs (*nuc*1 and *nuc*2). Notably, the presence of bands in the electropherogram other than the target bands was considered a primer dimer caused by protein denaturation because of multiple enzymes in the MIRA system.

**Table 3:** *Staphylococcus aureus-*MIRA primers used in this study.

Prime	Sequence (from 5′ to 3′)
Nuc-F	GTATGGCAATTGTTTCAATGTTACTTATAGG
Nuc-R	GTGTATCAACCAATAATAGTCTGAATGTCAT
Probe	CACAAACAGATAACGGCGTAAATAGAAG/i6FAMdT/G/idSp//iBHQ1dT/TCTGAAGATCCAAC

### Experimental optimization of temperature and time for MIRA

Amplification efficiency was highest at 40°C and lowest at 37°C (Figures 1C). The amplification efficiency increased with increasing temperature up to 40°C, then started to decrease, possibly because higher temperatures denature enzymes, thus reducing their activity in the multi-enzyme system. The final amplification curve (Figure 1D) showed that the reaction was completed in 20 min under the same fluorescence intensity, after which the amplification endpoint did not change. Nevertheless, the whole reaction was stable.

### Validation of *Staphylococcus aureus*-MIRA reaction product

**Figure 2:**
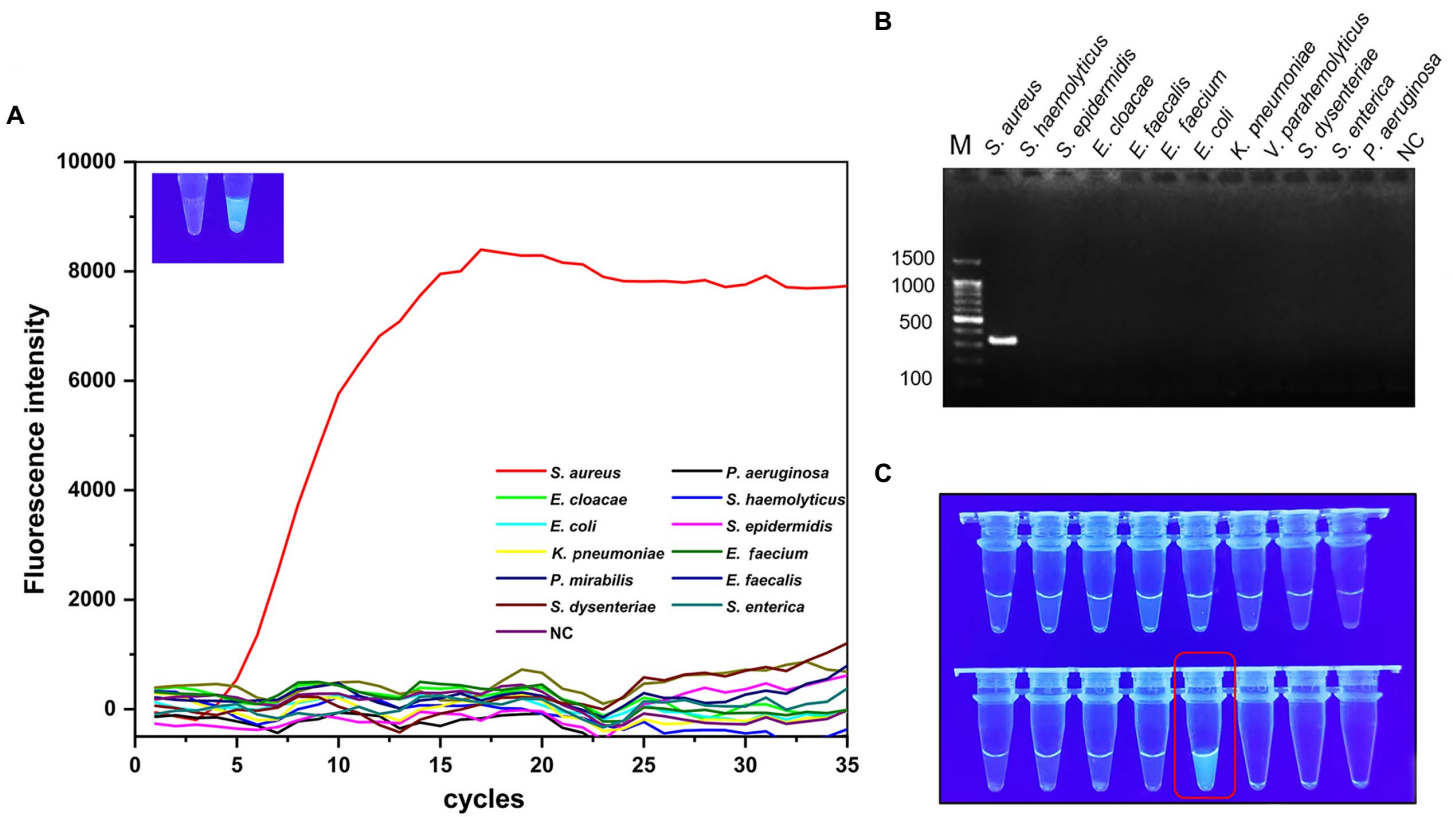
Specificity validation of MIRA. 12 strains were used for specificity verification through MIRA assay **(A)** and PCR assay **(B)**, the amplification curve and electrophoresis band were positive results, 11 non-*Staphylococcus aureus* strains and negative controls did not show amplification curves and target electrophoresis bands. **(C)** Visual verification of MIRA reaction products, red boxes marked positive results. NC, negative control; M, maker.

The products of *S. aureus-*MIRA amplification obtained at 40°C for 20 min were analyzed using agarose gel electrophoresis to validate the MIRA-based reaction system. The target band appeared in *S. aureus,* and the rest of the lane was not amplified (Figure 2B). The green fluorescence was detected with a naked eye under UV light (302 nm) for test tubes with *S. aureus* (Figure 2C). These findings suggest that the *S. aureus-*MIRA primer and reaction system was effective and specific for the detection of the target gene.

### Specificity verification

Only the target strain of the 12 strains showed a clear amplification curve (Figure 2A), indicating the assay had a high specificity. The target band was observed on the lane of *S. aureus* after agarose gel electrophoresis experiments (Figure 2B). However, the rest of the lanes did not have corresponding bands and no significant cross-reactivity with other strains. Additionally, strong green fluorescence was observed at 302 nm UV only in the reaction solution containing *S. aureus* (Figure 2C).

### Limit of detection analysis

**Figure 3:**
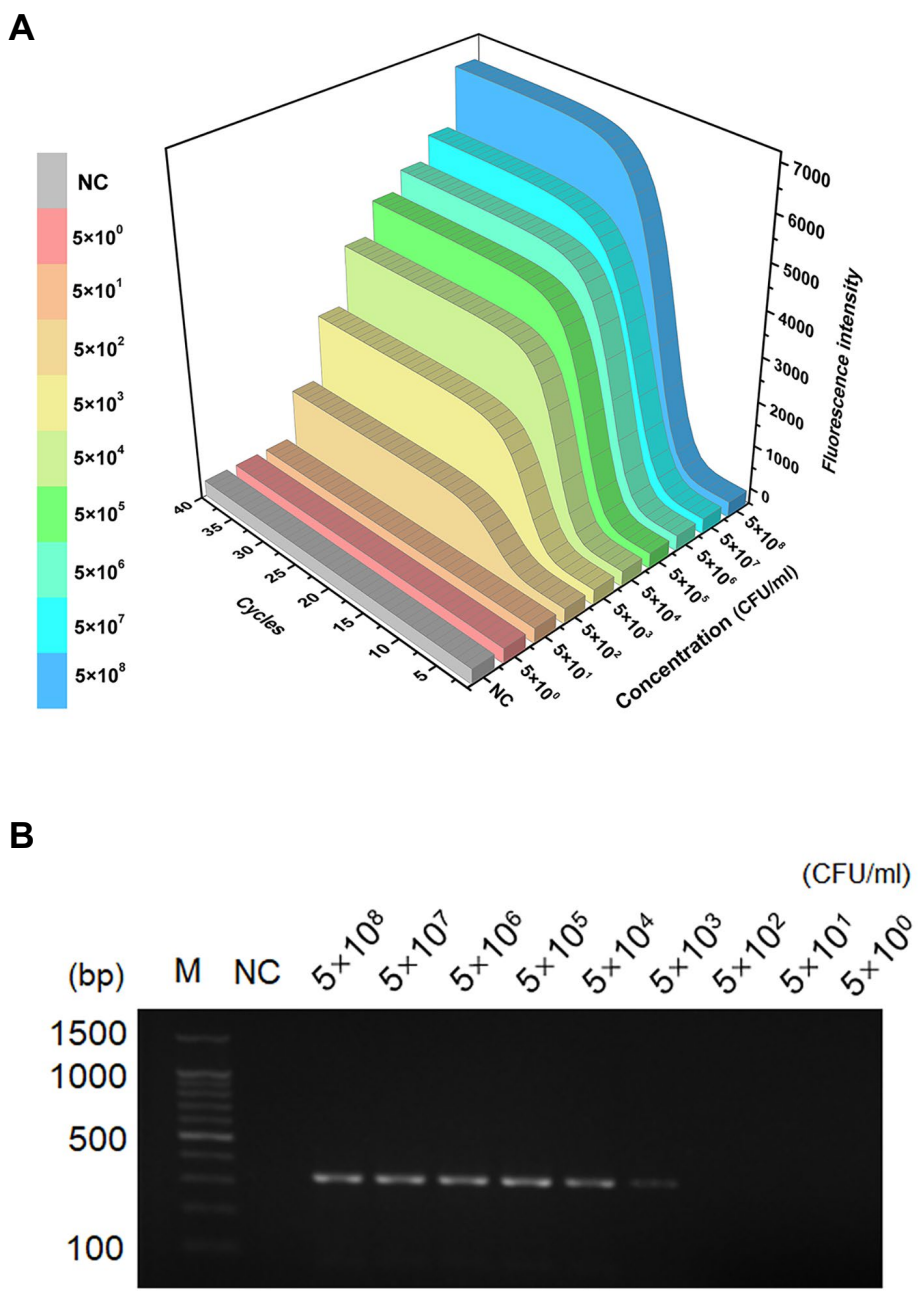
LOD of the LAMP and PCR assays. Sensitivity detection of genomic DNA of *S. aureus* by MIRA assay **(A)** and PCR **(B)**, The template levels from 5.0 × 10^8^ to 5.0 × 10^0^ CFU/ml. NC, negative control; M, maker.

The sensitivity and dynamic range of the MIRA assay were evaluated using genomic DNA from *S. aureus* standard strain based on optimal reaction conditions. The fluorescence intensity decreased with decreasing concentration of DNA template (Figure 3A). The amplification curve was invisible when the concentration was below 5 × 10^2^ CFU/ml. The limit of detection of PCR was 5 × 10^3^ CFU/ml. The agarose gel electrophoresis images (Figure 3B) showed faint bands when the concentration was 5 × 10^3^ CFU/ml. The limit of detection of MIRA (5 × 10^2^ CFU/ml) was 10 times lower than that of PCR (5 × 10^3^ CFU/ml), indicating that MIRA has higher sensitivity than the traditional PCR method.

### Schematic mechanism of MIRA assay

**Figure 4:**
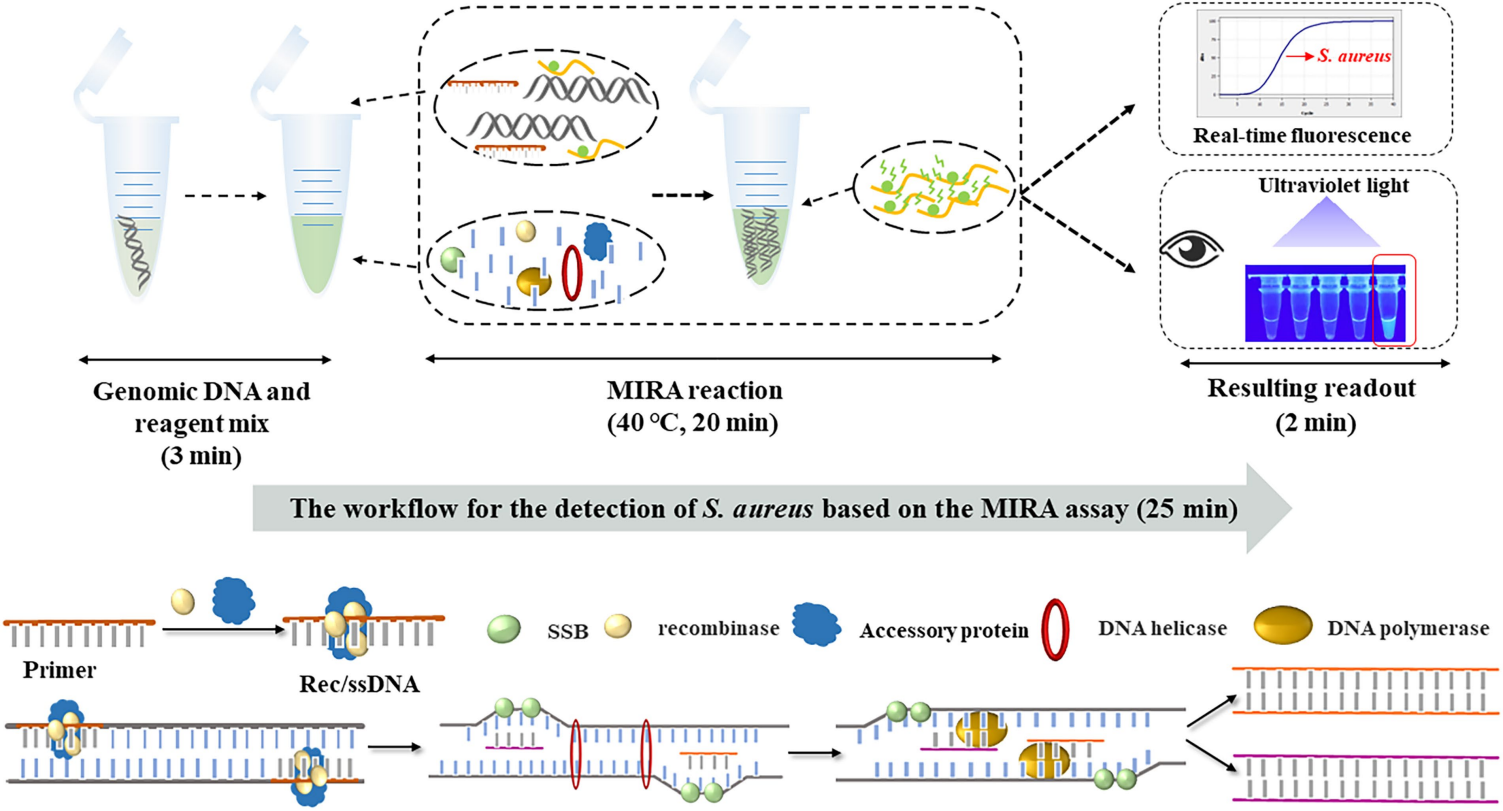
The workflow and schematic mechanism of *S. aureus* based on MIRA. *S. aureus*-MIRA assay contains three steps: mixing of genomic DNA and reaction reagents, MIRA reaction, observation of test results. The whole diagnosis procedure can be completed in 25 min.

The workflow and schematic mechanism of *S. aureus* based on MIRA are shown in Figure 4. In summary, the extracted *S. aureus* genomic DNA and MIRA reagent were put into the same test tube (3 min) and shook to mix. The sample was amplified at 40°C for 20 min. Several FAM-labeled fluorescent probes were freed after the amplification of the target gene, thereby emitting fluorescence. Finally, the fluorescent probes labeled with FAM were detected using qRT-PCR. The workflow for MIRA-based detection of *S. aureus* was completed in 25 min.

### Clinical efficacy of MIRA and analysis of test results

In this study, the practical application potential of the MIRA method was analyzed using 335 clinical samples. A total of 114 non-*S. aureus* strains did not test positive (specificity; 100%), indicating that the MIRA method has a high specificity (Table 4). However, PCR amplified one non-positive strain (specificity; 99.13%). Compared with the traditional culture method for clinical detection of *S. aureus*, MIRA had a similar specificity, while PCR had slightly less specificity. Moreover, MIRA and PCR did not positively detect only two strains of *S. aureus* (urine and sputum specimens; sensitivity; 99.10%). MIRA and PCR are nucleic acid molecular amplification technique both of which are analyzed with the same data in terms of sensitivity. Finally, the accuracy of MIRA and PCR tests was 99.4 and 99.11%, respectively. The higher specificity in MIRA may be related to the enzymes and cofactors in the MIRA system. After optimization and specificity validation of the MIRA assay method. 335 clinical specimens verified its feasibility with high accuracy. These results indicate that MIRA is fast and easy to use with high specificity and detection efficiency.

**Table 4:** Clinical efficacy testing by MIRA and PCR.

Method	Sensitivity (%)	Specificity (%)	Test accuracy (%)
MIRA	99.10%	100%	99.40%
PCR	99.10%	99.13%	99.11%

The results of the VITEK 2 compact system were employed as reference diagnostic results and were applied to the evaluation of the diagnostic efficiency of MIRA and PCR assays.

## Discussion

The current diagnostic methods for detecting *S. aureus* are either time-consuming (microbial culture; Hulme, 2017) or have a complex operation (PCR; Velasco et al., 2014). MIRA is a rapid and simple molecular diagnostic method that can rapidly diagnose pathogens. MIRA also greatly shortens the detection time of *S. aureus*. MIRA reaction takes 20 min, and the detection of the entire result (sample mixing, observation of results) takes 25 min. Besides reducing detection time from 2 to 5 days to 25 min, MIRA also eliminates many cumbersome steps associated with the traditional culture methods (Zhao et al., 2016). In this study, MIRA had a higher specificity and speed of detection than the traditional methods (microbial culture and PCR). MIRA is also in line with the concept of point-of-care testing bringing the test easily and quickly to the patient (Dhiman et al., 2017).

The specificity assay demonstrated that the primer could specifically distinguish *S. aureus* from other common clinical bacteria. Furthermore, an intense green fluorescence of 302 nm UV was observed only in the reaction solution containing *S. aureus*, indicating a high efficiency of *nuc* gene amplification. The optimum reaction temperature and time were 40°C and 20 min, respectively. MIRA had a lower reaction temperature and shorter detection time than several reported LMAP assays. The limit of detection of MIRA was 500 CFU/ml, which was 10 times lower than that of the conventional PCR. Moreover, MIRA successfully identified all non-*S. aureus* strains (specificity; 100%). The higher specificity in MIRA may be related to the enzymes and cofactors in the MIRA system. The modification of enzymes (targeted manner) in the MIRA system and screening of cofactors make MIRA to be more resistant to interference and more stable. However, two of 221 *S. aureus* strains were not positively detected using MIRA (sensitivity; 99.10% and detection accuracy; 99.40%). Besides observation using the qRT-PCR instrument, MIRA results were qualitatively analyzed under UV light at 302 nm, with positive results producing intense green fluorescence. Therefore, the whole experiment could be completed under simple conditions.

Many isothermal amplification-based methods have been used to detect *S. aureus* (Peker et al., 2018), including a single-tube RPA-CRISPR/Cas12 rapid detection platform (Liu et al., 2022) and a rapid detection method for loop-mediated isothermal amplification (Deng et al., 2019). However, MIRA enhances the speed, resistance to interference, and stability since it uses a different source of recombinant enzyme (*Streptomyces azure* recA, SC-recA) in a multi-enzyme system compared with RPA (T4 UvsX; Awate and Brosh, 2017) and RAA (*E. coli* UvsX; Geng et al., 2018). MIRA method has been widely used to detect many viruses and bacteria. However, lateral flow dipsticks (LFDs; Jiang et al., 2020), used for interpreting MIRA results, can complicate the findings by producing false positives since aerosol contamination can occur when the tube cap is opened. In this experiment, real-time fluorescence and UV light were used to interpret MIRA results to avoid this problem. In addition, a strong visible fluorescence was observed at 302 nm under UV light after the target gene was amplified because of the inclusion of a fluorescent-labeled probe. These results indicate that this assay can be further improved. Furthermore, this method had high efficiency and specificity.

In this study, MIRA is superior to PCR based on diagnostic effectiveness. MIRA is a fast and easy-to-use method with high specificity and detection efficiency and thus may be suitable for the rapid detection of *S. aureus* in clinical samples, particularly for routine diagnostic and infection control purposes. In the current molecular diagnosis, PCR, RPA/RAA, LAMP and other detection techniques all need to detect microorganisms within a certain detection limit. Similarly, since the concentration of *S. aureus* in clinical samples was very low and below the detection limit of MIRA, it could not be detected directly from the samples, which will be considered for further study in the future.

## Conclusion

In summary, MIRA method was developed and optimized for the detection of *S. aureus*. The limit of detection of this molecular technique MIRA method was 10 times lower than that of the conventional PCR and had a higher sensitivity (100% vs. 99.13%). MIRA significantly reduced the detection time and had a clinical diagnostic accuracy of 99.4%. The detection of *S. aureus* in the workflow took only 25 min. This study provides a rapid and simple method for the detection of *S. aureus* crucial for the early and rapid diagnosis of *S. aureus* infection.

## Data availability statement

The datasets presented in this study can be found in online repositories. The names of the repository/repositories and accession number(s) can be found in the article.

## Author contributions

PH: conceptualization, funding acquisition, investigation, supervision, writing original draft, and writing – review and editing. JL: investigation, visualization, writing original draft, and writing – review and editing. ZS: investigation and resources. CW: data curation and visualization. XY: resources. YH: conceptualization, investigation, project administration, writing – review and editing, and supervision. All authors contributed to the article and approved the submitted version.

## Funding

This study was financially supported by Innovation Team and Talents Cultivation Program of National Administration of Traditional Chinese Medicine (Grant No. ZYYCXTD-D-202209).

## Conflict of interest

The authors declare that they have no known competing financial interests or personal relationships that could have appeared to influence the work reported in this paper.

## Publisher’s note

All claims expressed in this article are solely those of the authors and do not necessarily represent those of their affiliated organizations, or those of the publisher, the editors and the reviewers. Any product that may be evaluated in this article, or claim that may be made by its manufacturer, is not guaranteed or endorsed by the publisher.
